# Optical Biosensors and Applications

**DOI:** 10.3390/s26041104

**Published:** 2026-02-08

**Authors:** Esmaeil Heydari, Hossein Zare-Behtash

**Affiliations:** 1 Nanophotonic Sensors and Optofluidics Laboratory, Faculty of Physics, Kharazmi University, Tehran 15719-14911, Iran; 2 Faculty of Engineering, Emirates Aviation University, Academic City, Dubai 507038, United Arab Emirates

## 1. Introduction

Optical biosensors constitute a category of analytical devices that capitalize on the interactions between light photons and biological elements to sense and quantify target analytes. Their significance lies in facilitating rapid, sensitive, contactless, and often label-free detection, which is crucial for real-time monitoring. A core principle of these devices involves transducing a biological recognition event into a measurable optical signal. This signal can manifest as a change in absorbance, photoluminescence intensity and lifetime, or refractive index. The applications of these sensors are vast and critically important across multiple fields. In healthcare, they represent a promising option for point-of-care diagnostics, enabling the rapid detection of pathogens, biomarkers, and viruses, and therefore facilitating immediate clinical decision-making. Within the food safety industry, they act as essential tools for the monitoring of chemical contaminants like histamine and heavy metals, as well as of pathogenic bacteria, contributing to consumer protection. Their ability to detect pollutants and toxins with high specificity also aids in environmental monitoring. Additionally, their compatibility with microfluidic systems facilitates the development of compact, portable Lab-on-a-Chip devices. This type of integration minimizes reagent use, automates sample handling, and allows for highly multiplexed analyses, making sophisticated diagnostic power accessible outside traditional laboratory settings.

## 2. Photonic Multi-Sensing Systems

The core innovation in this study lies in the synergistic combination of two advanced technologies: molecular gates for specific biorecognition and localized surface plasmon resonance (LSPR) structures for optical transduction [1]. Molecular gates are engineered materials consisting of a porous substrate loaded with a reporter molecule and capped with a receptor. The presence of the target analyte triggers the gate to open and release the reporter. This release causes a significant local change in the refractive index, which is detected by the adjacent LSPR probe, acting as a powerful signal enhancement mechanism. This system is designed to be multiplexed, and is capable of detecting up to 12 different analytes—including respiratory viruses (IVA, IVB, SARS-CoV-2, and RSV), chemical contaminants (methylmercury and histamine), and a foodborne pathogen (*Listeria monocytogenes*)—in a single test within a duration of approximately 30 min. The platform is conceived as a user-friendly, modular system comprising a disposable microfluidic cartridge including the sensor and a portable readout unit. This unit features a custom optical system that avoids the need for complex and expensive components like optical fibers. The project presents significant advantages over current methods like PCR and HPLC, including quicker results, lower costs per test, portability for on-site use, and comparable or superior sensitivity and selectivity, supported by validation against gold-standard techniques.

## 3. Optical Oxygen Sensing Based on Phosphorescence Quenching

This study presents a comparison between two instruments for dissolved oxygen detection: the traditional electrochemical Clark electrode and an optical sensor based on phosphorescence quenching [2]. The need for this comparison arose from the ongoing competition between the two technologies, each having its distinct advantages and drawbacks; these are typically difficult to assess as existing research has usually employed only one method. The authors designed a unique microfluidic system that enables both sensor types to measure oxygen levels in the exact same microvolume simultaneously, ensuring that experimental conditions are identical. A bioreceptor composed of *Saccharomyces cerevisiae* yeast cells adsorbed onto a specialized fluorinated composite material was used to consume oxygen in response to glucose samples. The results demonstrated that the optical sensor responded more quickly to changes in oxygen concentration, which was attributed to the absence of the diffusion-limiting membrane required by the Clark electrode. When applied for the estimation of the biochemical oxygen demand (BOD_5_) of real water samples, the optical sensor’s readings showed a near 1:1 correlation with the standard reference method, while the Clark electrode consistently produced underestimated values, particularly at lower concentrations. This study concludes that under the controlled, constant-flow conditions of a microfluidic system, the optical method offers superior metrological performance and response time, and requires less maintenance; thus, the latter is a more suitable and appealing transducer for whole-cell biosensing applications.

## 4. Optical Glucose Sensors Based on Photoluminescent Nanomaterials

This study details the development of a highly sensitive optical biosensor for detecting low levels of glucose, which relevant for the monitoring of both hypoglycemia and diabetes [3]. The sensor utilizes chitosan-capped manganese-doped zinc sulfide (CH-ZnS/Mn) nanomaterials, the photoluminescence of which is quenched in the presence of glucose. Chitosan serves as a biocompatible capping agent that stabilizes the nanoparticles and enhances their optical properties. This research systematically investigated the effect of different chitosan concentrations (0.75% to 1.5% wt), identifying 1% wt as optimal as this yielded the highest sensitivity and most stable performance. The sensor operates effectively in a very low glucose concentration range of 0.125 mM to 0.636 mM (2.3 to 11.4 mg/dL), with a detection limit of 0.125 mM, which is far below the clinical hypoglycemia threshold of 3.9 mM (70 mg/dL). Performance was validated not only in pure water but also in phosphate-buffered saline, a common buffer that mimics biological media in which the sensor maintained excellent linearity and sensitivity. This mechanism hinges on the quenching of the material’s photoluminescence intensity at 530 nm upon excitation at 365 nm as the glucose concentration increases. This work underscores the potential for the use of these nanomaterials in the development of cost-effective, rapid, and reliable diagnostic tools for monitoring glucose in saliva or other bodily fluids, opening up an avenue for the development of non-invasive point-of-care testing devices.

## 5. Noninvasive Optical Glucose Biosensing Based on Solid-State Laser Spectrometers

This article investigates noninvasive optical glucose sensing based on solid-state laser-based near-infrared spectrometer to address long-standing challenges in diabetes management [4]. The results of the proposed solid-state laser platform were compared to those obtained using a Fourier-transform (FT) spectrometer. Interestingly, the SECV values were 7.82 and 6.62 mg/dL for the two datasets used, respectively. Analysis showed that the SNR for the solid-state laser system was half that of the FT spectrometer results, thus representing a tangible platform for future design improvements. Overall, this work establishes the analytical utility of the proposed solid-state system, therefore justifying its further development, refinement, and testing in more complex matrixes, as well as their translational potential in clinical practice.

## 6. Optical Particle Tracking in Microfluidic Devices

This paper addresses the challenge of characterizing mixing performance in microfluidic devices, especially for complex scenarios with more than two input reagents [5]. The authors propose a novel methodology that features particle tracking, which is used as an alternative to traditional dye-based intensity measurements, in order to quantify mixing efficiency. They developed and implemented a free, open-source mobile application called MIQUOD for Android devices which can analyze both concentration field data (from dyes) and particle-tracking data. This application calculates eleven different performance metrics, including the mixing index, scale of segregation, and index of dispersion. A key contribution of this study is its proposal of a new mixing index for particles (m_p_) that normalizes data to allow for comparisons between different devices and experimental conditions. This method was tested numerically via COMSOL Multiphysics 5.4 simulations, as well as experimentally on several passive micromixer designs (Y-shaped, asymmetric split-and-recombine (ASAR), and a 3D sinusoidal mixer), using different-colored fluorescent microparticles. The 3D sinusoidal mixer showed the best performance (M = 88.7%), followed by the ASAR and Y-shaped designs. This study demonstrates that particle tracking, enabled by the mobile application, provides a more robust and descriptive way to assess mixing, particularly for multi-inlet systems, and represents a practical, accessible tool for optimizing microfluidic device design without relying on expensive software or complex setups.

## 7. Optical Biosensor Optimization of Surface Functionalization

This study focuses on optimizing surface functionalization strategies with which microring resonator (MRR)-based biosensors can enhance biomarker detection in liquid biopsy applications [6]. The authors compare two organosilanes—epoxysilane (GPTMS) and mercaptosilane (MPTMS)—for covalent aptamer immobilization. The key steps include plasma activation, silanization, aptamer binding, and passivation. Argon plasma and 1% MPTMS yielded the most homogeneous and reactive surfaces. Aptamer concentration and immobilization time were optimized to 1 µM and 3 h, respectively, with passivation using mercaptohexanol (MCH). The protocol was validated on photonic chips, demonstrating specific thrombin detection with minimal non-specific binding. The functionalized surfaces showed good stability and reproducibility, supporting their potential use in point-of-care biosensors.

## 8. Optical Sensing Based on Porous Silicon–Tamm Plasmon Structures

This study presents the development of a porous silicon-based multilayer device coupled with Tamm plasmon resonance for the sensitive sensing of cytotoxin-associated antigen A (CagA) of the bacterium *H. pylori* [7]. The proposed structure leverages the sharp optical resonance of Tamm plasmons at the interface between a dielectric Bragg reflector and a thin metallic layer. The simulation and experimental results demonstrate enhanced sensitivity compared to that of conventional porous silicon sensors. The authors report label-free detection, with a sensitivity of 100 pm/(ng/mL) and a limit of detection of 0.05 ng/mL. This research further explores the robustness and tunability of the structure by modifying porosity, layer thickness, and the metal coating. Such designs hold significant value for creating compact, low-cost, and highly sensitive biosensors that are suitable for real-time medical diagnostics.

## 9. Portable Biosensor Based on Gold Nanoparticles

This study presents a portable, gold-nanoparticle-based plasmonic biosensor for the rapid detection of carbapenem-resistant bacteria in water samples [8]. Targeting three key resistance genes (blaNDM-1, blaOXA-1, and blaKPC-3), the biosensor uses dextrin-capped gold nanoparticles functionalized with gene-specific DNA probes. Upon target binding, nanoparticle aggregation is prevented, resulting in a visible color change from blue to red. Detection is achieved through the use of spectrophotometry or a smartphone RGB application. A simple boiling method is used to extract DNA, and a portable thermal cycler enables field-based hybridization. Magnetic nanoparticles pre-concentrate bacteria from complex matrices like river water and turkey rinse. The biosensor detected genes at concentrations as low as 2.5 ng/μL, corresponding to ~10^3^ CFU/mL, without requiring PCR amplification. It successfully differentiated target from non-target bacteria across all sample types, demonstrating potential for point-of-care antimicrobial resistance surveillance in low-resource settings.

## 10. Summary and Conclusions

These papers highlight the rapid evolution and expanding capabilities of optical biosensing technologies, highlighting a clear trend toward their integration, multiplexing, and point-of-care applicability. The PHOTONGATE project exemplifies a top–down approach to creating a comprehensive, multi-analyte diagnostic platform by innovatively combining molecular gate chemistry with LSPR photonics to achieve unparalleled specificity and signal amplification. In contrast, the study on optical oxygen sensors provides a critical bottom–up, comparative evaluation of fundamental transduction methods, rigorously demonstrating the superiority of optical sensing over established electrochemical techniques in a controlled microfluidic environment for a specific application (whole-cell biosensing). The work on noninvasive and solid-state laser-based glucose monitoring tackles one of the most challenging biomedical sensing applications, highlighting both the technical obstacles and advances offered by optical and computational methods. Taken together, these studies demonstrate how optical biosensors can be transformed from proofs of concept into viable solutions by harnessing photonics, and advanced data analysis tools. The work on chitosan-capped nanomaterials focuses on the material science frontier, engineering nanoparticles for extreme sensitivity in detecting a fundamental analyte (glucose) at clinically relevant low concentrations, showcasing the potential for non-invasive monitoring. Despite the remaining hurdles of calibration, standardization, and manufacturing costs, the outlook for optical biosensors is highly promising. These technologies will not only improve diagnostics and disease management but also expand into environmental and industrial applications, highlighting their interdisciplinary impact. The research on particle tracking and the MIQUOD app tackles a crucial engineering challenge in microfluidics itself—precise performance characterization—by providing an open-source, accessible tool that enables the optimization of the very devices that will house these advanced biosensors. A unifying theme between these studies is the indispensable role of microfluidics in miniaturizing assays, handling samples, and enhancing sensor performance. The advancements presented—from novel biorecognition elements and transducer designs to new characterization methods—converge to push the entire field toward more powerful, affordable, and user-friendly analytical systems for healthcare, food safety, and environmental monitoring. The optimized functionalization protocol using MPTMS silanization and thiolated aptamers achieved high-density, oriented immobilization. Argon plasma effectively cleaned and activated surfaces. Ellman’s assay confirmed a high thiol density (~4 × 10^14^/cm^2^). Fluorescence and confocal microscopy verified the homogeneity of the aptamer coating. XPS and AFM characterized chemical and morphological changes. Specific thrombin detection was demonstrated, with significant signal differentiation between specific and non-specific aptamers. Passivation with MCH reduced non-specific binding. The protocol proved stable for up to two months at 4 °C. This work provides a robust foundation for developing sensitive, label-free biosensors for clinical diagnostics. The work on nanostructured porous silicon–plasmonic systems demonstrates its promise for highly sensitive and label-free biomolecular detection, bridging the gap between laboratory prototypes and scalable devices. In the research on high specificity detection of three carbapenem resistance genes in inoculated water, the limits of detection of the biosensor were 2.5 ng/μL for blaKPC-3 and 0.625 ng/μL for blaNDM-1 and blaOXA-1. Magnetic nanoparticles captured efficiently concentrated bacteria from various water matrices, though efficiency varied by strain and sample type. Zeta potential measurements revealed surface charge differences affecting capture efficiency. Smartphone RGB analysis results correlated well with spectrophotometric results, supporting field applicability. The system offers a rapid, low-cost, and portable alternative to conventional PCR for environmental AMR monitoring, thus being suitable for use in resource-limited areas.
